# Establishing Research Strategies, Methodologies and Technologies to Link Genomics and Proteomics to Seagrass Productivity, Community Metabolism, and Ecosystem Carbon Fluxes

**DOI:** 10.3389/fpls.2013.00038

**Published:** 2013-03-19

**Authors:** Silvia Mazzuca, M. Björk, S. Beer, P. Felisberto, S. Gobert, G. Procaccini, J. Runcie, J. Silva, A. V. Borges, C. Brunet, P. Buapet, W. Champenois, M. M. Costa, D. D’Esposito, M. Gullström, P. Lejeune, G. Lepoint, I. Olivé, L. M. Rasmusson, J. Richir, M. Ruocco, I. A. Serra, A. Spadafora, Rui Santos

**Affiliations:** ^1^Department of Chemistry and Technology, University of CalabriaRende, Italy; ^2^Department of Ecology, Environment and Plant Sciences, Stockholm UniversityStockholm, Sweden; ^3^Department of Plant Sciences, Tel Aviv UniversityTel Aviv, Israel; ^4^LARSyS, University of AlgarveFaro, Portugal; ^5^Département de Biologie, Ecologie et Evolution, MARE, Université de LiègeLiège, Belgium; ^6^Stazione Zoologica Anton DohrnNaples, Italy; ^7^School of Biological Sciences, University of SydneySydney, NSW, Australia; ^8^Marine Plant Ecology (ALGAE), Center of Marine Sciences, University of AlgarveFaro, Portugal; ^9^Chemical Oceanography Unit, Université de LiègeLiège, Belgium; ^10^STARESO SAS, Pointe RevellataCalvi, France

**Keywords:** seagrasses, proteomics, genomics, carbon fluxes, photosynthesis, respiration, productivity, marine

## Abstract

A complete understanding of the mechanistic basis of marine ecosystem functioning is only possible through integrative and interdisciplinary research. This enables the prediction of change and possibly the mitigation of the consequences of anthropogenic impacts. One major aim of the European Cooperation in Science and Technology (COST) Action ES0609 “*Seagrasses productivity. From genes to ecosystem management*,” is the calibration and synthesis of various methods and the development of innovative techniques and protocols for studying seagrass ecosystems. During 10 days, 20 researchers representing a range of disciplines (molecular biology, physiology, botany, ecology, oceanography, and underwater acoustics) gathered at The Station de Recherches Sous-marines et Océanographiques (STARESO, Corsica) to study together the nearby *Posidonia oceanica* meadow. STARESO is located in an oligotrophic area classified as “pristine site” where environmental disturbances caused by anthropogenic pressure are exceptionally low. The healthy *P. oceanica* meadow, which grows in front of the research station, colonizes the sea bottom from the surface to 37 m depth. During the study, genomic and proteomic approaches were integrated with ecophysiological and physical approaches with the aim of understanding changes in seagrass productivity and metabolism at different depths and along daily cycles. In this paper we report details on the approaches utilized and we forecast the potential of the data that will come from this synergistic approach not only for *P. oceanica* but for seagrasses in general.

## Introduction

Numerous challenges can frustrate interdisciplinary research. One problem that often occurs with interdisciplinary projects is scoping the research problem. For example, it is impossible for a single person or laboratory to possess the range of skills needed to conduct truly interdisciplinary research on seagrasses. With this study, we may not have achieved many of our findings and our collective understanding would have been far less refined if we were not engaged in interdisciplinary research. Hence, researchers need to embrace collaboration with colleagues in other disciplines, such as functional genomics, proteomics, ecology, conservation, and physiology (Boudouresque et al., 2009). We anticipate that such synergies as have been outlined below will stimulate advances in other areas of seagrasses, similar to those we have been able to accomplish on *Posidonia oceanica* (L.) Delille. Such interdisciplinary programs are not difficult to launch because stakeholders often have shared experiences and shared concepts. However, working with colleagues who are outside of one’s normal peer group can present challenges, particularly with respect to becoming fluent with the methodological basis and the scientific and technological limitations of the different specialties. *A high open-mindedness for different research background as well as a reciprocal sense of confidence and regard can be helpful*. Obtaining funding for interdisciplinary research can also be challenging based on the organizational structure of granting/funding agencies as well as the institutional structure of a team that may undertake the research. For example, our team consists of *academics* from several institutions as well as several research agencies. In our case, the crisis of seagrasses conservation and the need for coordinated research yielded support from the Cooperation in Science and Technology (COST) Action program of the European Science Foundation with flexibility in how funds could be disbursed to different team members.

Previous work has focused on (a) how to incorporate the comparative gene expression studies with photosynthetic performance, carbon and nitrogen utilization, and environmental adaptation, and (b) how to combine the research related to mechanisms of carbon utilization, light requirements, temperature effects, and natural variation in pH and ocean acidification (The Royal Society, 2005; Hall-Spencer et al., 2008; Arnold et al., 2012). This work concluded that we are not yet ready to comprehensively link these disciplines because the seagrass research community is still in the nascent stages of linking eco-physiology with genomic responses. In particular, the carbon and nitrogen metabolism of seagrasses have not yet been sufficiently well-studied and the genomics has only been able to assign meaningful interpretations to a few differentially expressed genes (Procaccini et al., 2012).

**Figure 1:**
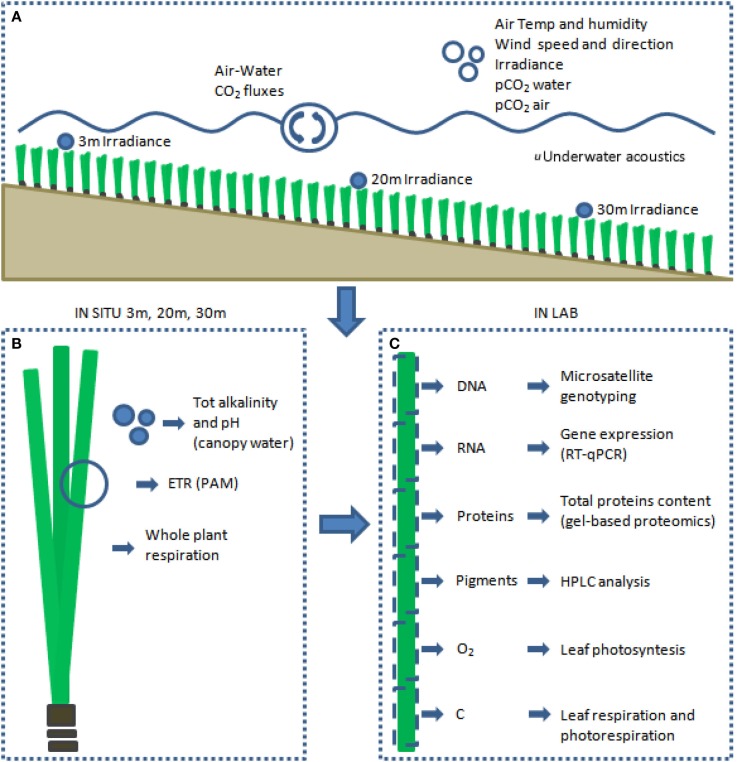
**Summary of methodological approaches performed *in situ* at community level (A) and at plant level (B)**. Replicate shoots were collected for each depth for physiological and molecular analyses that were performed all on the same leaf **(C)**.

Through the experimental design carried out at the Station de Recherches Sous-marine et Océanographiques (STARESO) we wished to fill these gaps and to create as links between observations at an individual and population level, and then scale up these links to the community/ecosystem level (Figure 1).

*Posidonia oceanica* covers about 2% of the seafloor (25,000–50,000 km^2^) in the Mediterranean Sea (Pasqualini et al., 1998). This endemic species grows to considerable depths, with meadows recorded from 0.5 to 40 m (Bouderesque and Meinesz, 1982), and living plants at 48 m depth. *P. oceanica* requires seawater of good quality, with low turbidity and a sedimentary budget compatible with the growth of the rhizomes and of the mat. *P. oceanica* is the most emblematic species of the Mediterranean; this robust phanerogam with long ribbon-shaped leaves grouped in clusters (shoots) is characterized by the prevalence of the asexual mode of reproduction (propagation through a dense web of plagiotropic rhizomes). In comparison with other seagrass species it is: long-lived (4–30 years) with long leaf life-span (70–350 days); the rhizome biomass shows low seasonal variability, the density (number of shoots per m^2^) is relatively constant throughout the year and from year-to-year (Gobert et al., 2006), its growth rate is extremely slow, and it forms highly productive meadows (Hemminga and Duarte, 2000). The biology and the ecology of this species are well known (Bouderesque and Meinesz, 1982; Boudouresque et al., 2006; Pergent et al., 2012). In 1999, one of every four papers on the biology and ecology of all seagrass species was devoted to *P. oceanica* (Duarte, 1999). Mediterranean meadows have been studied more than the other species. Measurements in *P. oceanica* beds have been carried out at different locations of the Mediterranean Basin, but usually dealt only with seagrasses themselves analyzed at very shallow depths with different methods, at different scale. Such data can hardly be extrapolated in order to estimate the importance of any entire *P. oceanica* meadow. Since 2000, the number of international papers on *P. oceanica* has considerably increased but there is still a paucity of papers with an interdisciplinary research focus.

What is the evolutionary potential of selected species to adapt to short and long-term environmental changes as imposed by human impacts on natural systems? The incorporation of genomic and transcriptomic techniques in the analysis of marine ecosystems and species can help us determine this (Procaccini et al., 2007; Reusch and Wood, 2007). Most of the more advanced-omics techniques have been developed in laboratory model-species, such as *Arabidopsis* or *Oryza*, but some of them are also applicable in species for which genomic resources are scarce or absent, such as most of seagrass species. In the last few years, seagrass genomic and transcriptomic resources are increasing, particularly in two species of the genus *Zostera*, *Zostera marina*, and *Z. noltii*, and on *P. oceanica*. An on line EST (Expressed Sequence Tags) database, Dr. Zompo (http://drzompo.uni-muenster.de/; Wissler et al., 2009), collects all *Z. marina* and *P. oceanica* ESTs available to date, but thousands of new expressed sequences are becoming available in the near future for both species, thanks to next generation sequencing approaches. The complete genome sequencing of *Z. marina* has been also performed, thanks to a JGI Community Sequencing Project – CSP 2009 (coordinator J. L. Olsen, University of Groningen, Netherlands).

Annotated EST libraries represent the starting point for a number of approaches relevant to molecular studies of ecological genetics of natural populations (Bouck and Vision, 2007). In seagrasses, recent papers address the adaptive response to environmental forcing, such as light and temperature, assessing gene expression by means of EST-related approaches. The response to temperature stress has been approached in *Z. marina* and *Z. noltii* through transcriptomic profiling and gene expression of target genes (Reusch et al., 2008; Bergmann et al., 2010; Franssen et al., 2011; Massa et al., 2011; Winters et al., 2011; Gu et al., 2012). Gene expression variation in response to light along a depth gradient is being examined in *P. oceanica* (Procaccini et al., 2010; Serra et al., 2012) while the comparative analysis of EST libraries has been performed for approaching evolutionary questions related to seagrass evolution (Wissler et al., 2011). Catalogs of expressed sequences also represent a source of putatively not-neutral markers that can be utilized for searching outliers related to environmental features. EST-linked microsatellites have been isolated both in *Z. marina* and *P. oceanica* (Oetjen and Reusch, 2007; D’Esposito et al., in press) and have been utilized, together with SNPs markers, in a genome-scan analysis on *Z. marina* (Oetjen et al., 2010).

Boosting genomics information in EST database makes, from now, proteomic analyses more attractive for *P. oceanica* than in the past, because protein sequence analysis and identification are less challenging.

Proteomics is a promising powerful tool to compare quantitative/qualitative differences in thousands of proteins in *P. oceanica* from meadows living in different environments. Hence, by identifying the expression of different proteins under various conditions, we might validate these proteins as early biomarkers for eco-physiology assessment. On the other hand, the various metabolic pathways which are utilized under different conditions could represent a starting point to clarify how *P. oceanica* is able to adapt. To express its full potential, proteomics must rely on samples of high protein quality. Consequently, the newer methods of extraction, separation, and analysis of the entire proteome from a specific tissue, or from organelles that are now evolving in many plants, must also be implemented for seagrasses at local and large scales. The protein expression approach and the bottom-up experimental design together with the high-throughput technologies for mass spectrometry promise a large amount of empirical information on the seagrass proteome in the coming years. This large amount of information should be added to relevant seagrass databases in order to facilitate the organization of data to generate testable hypothesis. The application of new technique(s) combining two- or one-dimensional (1D) SDS-PAGE with a high-mass-accuracy LC-ESI-MS and LC-SACI-MS and MS/MS to sequence identification approaches has demonstrated an increase in the confidence of results (Finiguerra et al., 2010). This can provide a high-throughput system to achieve the goal of sequencing complete proteomes from seagrass organs and tissues. By databases such as EST, Transcriptomics, and Genomics, the genomic data from seagrasses can be interrelated with the emerging protein sequences and metabolic data as well as with environmental information.

How are seagrasses able to biochemically survive a marine life style? Since the proteome of each living cell is dynamic, proteomics allows investigators to clarify if, and to what extent, various pathways are utilized under varying conditions and triggered by the action of the environment on the system, and the relative protein level response times. Previously, two-dimensional gel-based proteomic studies on *Posidonia* meadows acclimated to different light conditions revealed physiological pathways involved in the acclimation of seagrasses to low-light, evidenced by Rubisco down-regulation; in contrast, enzymes involved in carbohydrate cleavage (1-fructose-bisphosphate aldolase, nucleoside diphosphate kinase, and beta-amylase) were up-regulated (Mazzuca et al., 2009). Afterward, the 1D gel-based proteomics and label-free approach applied to shaded adult leaf tissues showed significant down-regulation of the isoforms of β-carbonic anhydrase (Serra and Mazzuca, 2011). This kind of high-throughput proteomics revealed also that about 40% of the differentially expressed proteins in low-light appeared to be involved in chloroplast metabolic pathways (Dattolo et al., submitted). The “sub-organelle proteomics” strategy from the three different compartments – envelope, stroma, and thylakoids (Ferro et al., 2010) – is now being applied to *P. oceanica* (Mazzuca, personal communication).

Genetics can provide the bases for the plant physiological response to different environmental forcing, because it can be more or less plastic at either individual, population or species level. Precise knowledge of population genotypic composition and population genetic isolation/connectivity with distinct populations can help in interpreting functional responses and in framing the results of functional studies. In order to do this, we used species-specific microsatellite markers to genotype a standard representative number of individuals collected at 5 and 20 m depth. On the same individuals, newly selected EST-linked microsatellite were also scored, in order to look for outlier loci, that could be linked to specific environmental variables.

The study has been conducted both at the community level and plant level. At the community level we aimed to estimate the net community production (NCP) and the community respiration (CR) of *P. oceanica* using incubation chambers and monitoring the evolution of O_2_ production and consumption, respectively. At plant level we aimed to understand the primary metabolic pathways involved in the carbon budget, from the expression of selected genes, to the expression of proteins, to the assessment of photosynthetic and respiratory performance. Key genes have been selected along the whole photosynthetic and respiratory pathway and their expression has been evaluated by RT-qPCR along daily cycles at different depths. The photosynthetic pathway includes genes showing positive selection in respect to terrestrial plants (Wissler et al., 2011), and is worth investigation. We used an RNA-Seq approach used to detect differentially expressed genes between depths and plant portions (leaves and roots). On the side of proteins we applied the 1D label-free approach coupled with a spectral counting strategy to look at the overall expressed proteins (Schulze and Usadel, 2010). Although we expected the overall pattern of protein expression to be similar to that of mRNA expression, the incongruent expression between mRNAs and proteins can occur, emphasizing the importance of posttranscriptional regulatory mechanisms in cellular development, or perturbations that can be unveiled only through integrated analyses of both proteins and mRNAs. Thanks to the quantitative proteomic techniques (1D electrophoresis and mass spectrometry), we evaluated the correlation of each selected mRNA at corresponding protein level. The aim has been to capture a meaningful variation of selected protein expression (up and down) that can overlap with the differential expression of mRNA (up or down).

Since photosynthesis is the basis for plant growth, it follows that there should be a correlation between photosynthesis and growth. There should be a positive correlation between the rate of photosynthesis during the daytime corrected for that of respiration dielly (during the day and the night) and growth rate. As long as this balance is positive, i.e., daily photosynthesis exceeds diel respiration, the plants should grow if not constrained by other, non-photosynthetic or non-respirational, influences (such as grazing or uprooting or the like). This correlation between photosynthesis + respiration and growth is easy to show for simply built plants such as micro- and macro-algae (e.g., Lipkin et al., 1986), but is much harder to quantify for higher plants such as angiosperms. In seagrasses, which like their terrestrial-plant counterparts have both above- and below-ground tissues, it is relatively easy to measure rates of photosynthesis and respiration of the leaves, but much harder to measure rates of respiration of the underground roots and rhizomes, and especially so when *in situ* rates are sought. Therefore, till now, rates of photosynthesis have been used as a general indicator of the growth status of seagrasses, but respiration has largely been ignored. In this study we incorporate this important factor when measuring whole-plant or plant- community-based metabolism as a proxy for seagrass growth.

Photosynthesis and respiration measurements have traditionally been based on either O_2_ or CO_2_ exchange. In the aquatic environment, O_2_ measurements are far easier to perform than those of CO_2_ exchange. The big advantage in using such gas exchange measurements as a proxy for plant growth is that results can be obtained quickly (minutes to hours, rather than days to weeks for growth measurements). During the past 10 years, an even quicker method has been developed for photosynthetic measurements with a resolution time of seconds to minutes: pulse-amplitude modulated (PAM) fluorometry. This method measures quantum yields (Y) as photosynthetic electron transport per photon absorbed by the photosynthetic pigments. When multiplying Y with the photosynthetic active radiation (PAR) absorbed by the photosynthetic pigments of photosystem II (PSII), then photosynthetic electron transport rates (ETR) can be calculated in mol electrons m^−2^ leaf surface s^−1^. It should also be noted that parameters indicating stress, as well as considerations of the mechanisms involved in photosynthesis and other non-photosynthetic processes (e.g., photosynthetic and non-photosynthetic quenching), can also be elucidated by PAM fluorometry. While the quantitative accuracy of this method has been verified for several (e.g., Beer et al., 1998), and especially thin-leaved (Beer and Björk, 2000) seagrasses, its main drawback is that it ignores respiration and, thus, only photosynthetic rates *per se* can be measured. In order to obtain time series of these photosynthetic measurements, modulated fluorometers have been developed that can measure photosynthetic parameters *in situ* continuously for several days (Runcie et al., 2009; Runcie and Riddle, 2012).

In recognizing that respiration must be included in metabolic measurements that lead to information regarding growth rates, we are now trying to incorporate such measurements, either *in situ* or in the laboratory while mimicking *in situ* conditions. Thus, the present consortium will complement other groups by providing diurnal data not only on photosynthetic rates, but on gas exchange in general and respiration in particular.

Finally, unlike *in situ* methods, which only provide local measurements of photosynthesis related parameters, acoustic based methods can potentially allow the instantaneous quantification of oxygen production at meadow level, giving an integral estimate of O_2_ concentration along the propagation paths of the acoustic signal. In general, acoustic signals propagating through the ocean are sensitive to gas bubbles (Wilson, 1988; Medwin and Clay, 1998). In previous experiments (Hermand and Alberotanza, 2000; Hermand, 2003), it was shown that signatures in acoustic signals transmitted through *P. oceanica* meadows were highly correlated with the photosynthetic rate, which was ascribed to produced bubbles and gas filled aerenchyma. Wilson et al. (2012) observed a similar correlation in an experiment conducted in a *Syringodium filiforme* meadow, but in this case at a plant shoot scale. The acoustic system, as a low cost remote sensing tool to assess the photosynthetic activity of the *P. oceanica* meadow was here used in real time, although a fully operational system requires further investigation in methods for system calibration.

Here, as first step for calibration we also used a mooring at 10 m with an array of three optodes for oxygen measurements at hourly intervals. The temporal changes at daily scale of water column inventory of O_2_ allows the quantification of GPP and CR based on the Odum (1956) method. This approach was used successfully at STARESO (Champenois and Borges, 2012).

## Methods and Strategies

### How important is the location in our approach

**Figure 2:**
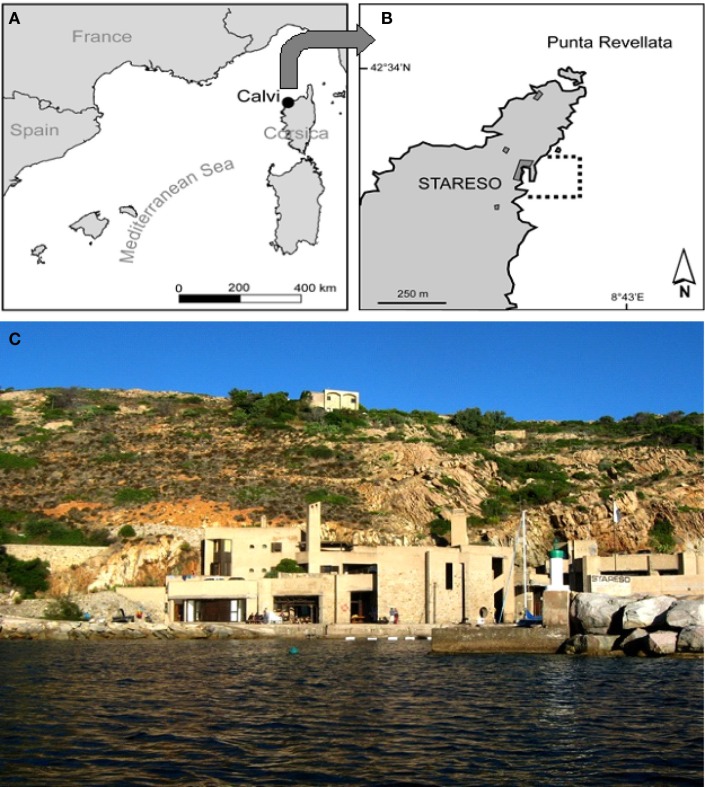
**Location of the study area in (A, B) the Calvi Bay, North Westerrn coats of Corsica, Mediterranean Sea, at the latitude and longitude of 8°450E, 42°350N. (C)** The Station de Recherches Sous-marines et Océanographiques, STARESO.

In the framework of marine interdisciplinary research, the site where field experiments are matched with lab activities is central. STARESO (8°45′E, 42°35′N) belongs to the University of Liège (Belgium) and acts also as a Technical Office toward communities and private customers in the field of marine environmental impact studies. STARESO is located in the Calvi Bay on the northwest coast of Corsica in the Mediterranean Sea. This oligotrophic area is classified as a “pristine site” where environmental disturbances caused by anthropogenic pressure are exceptionally low. The study site includes representatives of most major coastal ecosystems of the Mediterranean. The Calvi Bay is characterized by healthy benthic and pelagic ecosystems associated with a high biodiversity close the Liguro–Provençal current (Figure 2). The marine lab offers direct access to the sea, and facilitates investigations using diving, boats, and laboratories. Since 1970, time series of physical, chemical, and biological data (sampling at sea with automated systems and sensors deployed in the Bay, as well as *in situ* experiments) have been recorded. In front of the lab, *P. oceanica* (L.) Delile is the dominant ecosystem going from the surface to a lower limit that reaches 37 m.

A large collection of data focused on *P. oceanica* ecosystem diversity and functioning has been collected over the last 40 years. As a result, the seasonal and inter-annual dynamics of the major primary producers relating to the ambient parameters (temperature, winds, nutrient concentrations) are well known in the site (Bay, 1984; Lepoint et al., 2002; Gobert et al., 2003a). In spite of the very low nutrient concentrations, the meadow displays high biomass and productivity (more than 500 gdw m^−2^ y^−1^) and is considered to be a Low Nutrient-High Chlorophyll (LNHC) system (Gobert et al., 2002). The meadow is healthy (Gobert et al., 2009) and no significant changes of the vitality have been registered since 1975. Long-term follow-up show only classical interannual and seasonal variations of biomass and production that relate with ambient factors (temperature, winds, light) (Bay, 1984; Gobert et al., 2003a). However, an increase of the flowering frequency has been observed since 1975, and this may be related to the general increase of the temperature in the Mediterranean Sea (Gobert et al., 2001). Furthermore, local evidence of mechanical damage due to the anchoring of recreational boating has been recently detected.

The direct proximity of underwater *in situ* field analysis and the wet and dry lab allowed very easy sampling of biological material and fast processing of tissues for molecular analysis. Quality of the results due to this proximity is can be enhanced with continuous installation of different kind of *in situ* probes directly connected to the lab (e.g., salinity, temperature, weather station).

Finally, in the same way as an oceanographic ship, the marine lab offers full logistic capabilities (meeting rooms, efficient internet connection, meals, and lodging accommodations) that enabled scientific work day and night without interruption. As a consequence, one has confidence in results derived from the study, in particular those obtained through integration and interpretation of data from the different scientific disciplines.

### What timing, what methodologies and technologies for cooperative samples collection, *in situ* deployment of equipment, data collection, and analysis

As pointed out above, there are several ways to estimate the metabolic processes of seagrasses, each having its advantages, but measuring with different approaches. In order to compare such data, sampled with different methods, it is important to perform simultaneous field methods calibrations. We selected the meadow along a deep gradient and fixed daytimes corresponding to supersaturating and limiting irradiances as extreme conditions. Between these, many intermediate times were considered. Timing between the underwater *in situ* field analyses, the sampling of biological material and the processing of tissue for molecular analyses, which is typically done by fixing tissues in suitable buffers or by freezing them in liquid nitrogen, is the real challenge; the shorter the time between these events, the greater the confidence in the results from different specialties. To achieve this goal the sampling design has been careful planned in terms of the number of operators, suitable devices, and tools, time needed to carry each sample *from the sea to the lab* (Table 1).

**Table 1:** **Days, sampling time, sea depths, and suitable devices and tools for field measurement at seagrasses beads and for laboratory analyses on leaf biological replicates**.

TIME and place			Devices in field							Sampling of leaves for lab						
Days	Depth	Time	Shutter fluorometers/PAM	Classic fluorometers	Diving-PAM	RLCs Sensors	Minilog TR recorders	o_2_ Acoustic sensors	co_2_ Incubation chambers	Pigments	Epiphyte community (after ^13^C incubation)	o_2_ Electrodes	Genetics	RT-qPCR	lllumina RNA-seq	1-DE Free-label proteomics
From 12th to 17th October 2011	5 m	6:00				Every 15 min				Three biological replicates	10 Biological replicates			Three biological replicates		
		7:00														Two biological replicates
		9:00				Every 15 min							20 samples			Two biological replicates
		12:00				Every 15 min									Pulled leaves	
		13:00														Two biological replicates
		15:00				Every 15 min										Two biological replicates
		18:30				Every 15 min									Pulled leaves	
		0:00													

	20 m	6:00				Every 15 min				Three biological replicates				Three biological replicates		
		7:00														Two biological replicates
		9:00				Every 15 min							20 samples			Two biological replicates
		12:00													Pulled leaves	
		13:00									10 Biological replicates					Two biological replicates
		15:00				Every 15 min										Two biological replicates
		18:30				Every 15 min									Pulled leaves	
		0:00														Two biological replicates

	30 m	6:00				Every 15 min										
		7:00								Three biological replicates				Three biological replicates		Two biological replicates
		9:00				Every 15 min										
		12:00				Every 15 min											
		13:00								Three biological replicates	10 Biological replicates			Three biological replicates		Two biological replicates
		15:00				Every 15 min										
		18:30				Every 15 min									
		0:00													

Therefore, in this activity we set out to compare.

**Figure 3:**
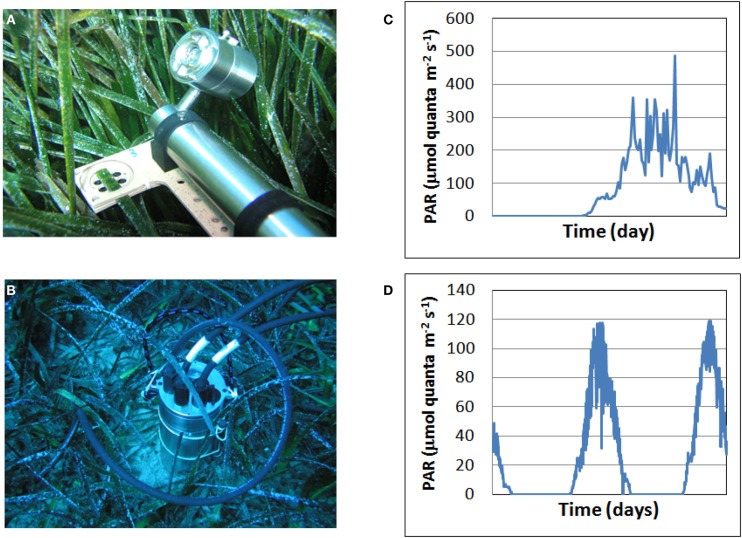
**Submersible modulated Shutter Fluorometer (A) and Submersible Datalogger (B), Aquation Pty Ltd, Australia and detected daily PAR measurements in shallow (C) and deep (D) plants**.

(i) How photosynthetic rates obtained by using modulated fluorometry may correlate with gas exchange measurements at both the plant and the community levels (O_2_ electrodes in the lab and community metabolism as well as modulated fluorometry *in situ*.
(ii) How continuous measurements with the autonomous modulated fluorometers correlate with the discrete measurements obtained with the conventional Diving-PAM.
(iii) How circadian changes in acoustic signal correlates with gas exchange measurements at the community levels.
(iv) How to catch the photosynthetic regulation change in relation to light intensity (shallow site and deeper site) during the day.
  To address these questions, submersible modulated fluorometers (Shutter Fluorometer and Classic Fluorometer, Aquation Pty Ltd., Australia, (Figure 3) were deployed for ∼24 h at 5, 20 and 30 m depth in the afternoon of the 16th October 2011. Seagrass leaves were positioned in the sample holders of the fluorometers so that a portion of leaf halfway along the blade was examined. Epiphytic material was gently removed by rubbing. After ∼24 h, new leaves were positioned in the sample holders and a further ∼24 h measurement was conducted. Leaves were oriented horizontally. Irradiance was measured both nearby with a dedicated light logger, and using the PAR sensor that is part of the shutter fluorometer.
  At 5 m depth, a total of four leaves were measured over the 2 day period. At 20 m depth, two individual leaves were measured, and at 30 m depth three leaves were measured each day, making a total of six leaves over the 2 day interval. Leaves were collected from 5 and 30 m depth and absorptance of these leaves was measured using a Diving-PAM light sensor calibrated against a LiCOR 193SA PAR sensor (Beer and Björk, 2000).
  One shutter fluorometer each at 5 and 20 m depth were programed to perform rapid light curves (RLCs) on samples at 06:00, 9:00, 12:00, 15:00, and 18:30 h. All fluorometers, including those programed to conduct RLCs, conducted effective quantum yield measurements every 15 min. In addition, every second measurement was followed by 10 s of exposure to far red light (FRL) with ambient light excluded using the shutter; this was followed by another saturating pulse measurement. From the measurement immediately following the FRL we determined Fo’, and used this value to calculate components of non-photochemical quenching (Runcie et al., 2009). Effective quantum yield measurements (excluding those immediately after exposure to FRL, or those obtained during a RLC) were used to calculate ETR, and diel PE curves were constructed by comparing ETR with ambient irradiance measured at the time of measurement. For these calculations we used absorptance values as obtained from leaves at 5 and 30 m; 20 m samples were assumed to be similar to those at 30 m (see Runcie et al., 2009). The value 0.5 was used, assuming equal sharing of exciton energy between Photosystems I and II. RLCs and diel PE curve data were described using models of Platt et al. (1980) with a term for photoinhibition or Webb et al. (1974) (two parameter model with no term for photoinhibition); non-linear least squares minimization techniques using the Levenberg–Marquardt algorithm were employed using the Optimiz software. Diel PE data were pooled for all leaves measured over the 2-day interval at each depth, and a single model fit to this data. Error values for Ek estimates were calculated by propagation of errors. Data are reported with means and standard errors. Non-photochemical quenching components were calculated as described in Runcie et al. (2009).
(v) How do long incubation times affect estimates of community metabolism?
  The effects of the duration of incubation on the estimations of community metabolic rates have been tested here for the first time. The rationale to test this is that the deployment of the incubation chambers over a dense seagrass meadow results both in the accumulation of O_2_ within the chambers and an increase in pH due to the photosynthetic consumption of CO_2_. At high O_2_ and low CO_2_ levels, the enzyme Ribulose-1,5-bisphosphate-carboxylase oxygenase switches from carboxylase to oxygenase activity (Heber et al., 1995). Under these conditions, there is consumption of O_2_ and release of CO_2_ by photorespiration, which will result in the underestimation of GPP. On the other hand, the CO_2_ photosynthetic consumption by seagrasses in closed environments may drive the pH to values up to 9.2 (Beer et al., 2006), causing a linear decrease of the photosynthetic rates (Invers et al., 1997). The availability of dissolved CO_2_ at high pH levels is residual and thus the photosynthetic production is only possible if producers are able to utilize the very abundant HCO_3_^−^ form of inorganic carbon. Even though many marine macrophytes, including seagrasses, have been found to be able to utilize HCO_3_^−^ as an external source of inorganic carbon for their photosynthetic needs (Beer et al., 1998), the rate of CO_2_ consumption will be lower and thus the GPP will be underestimated.
(vi) How to evaluate the contribution of epiphytic communities on the *P. oceanica* leaves to the overall C-flux?

The parallel use of two techniques has been implemented to evaluate the contribution of epiphytes living on *P. oceanica* leaves to NCP: “^13^C tracer incorporation” and “Biomass accumulation.” ^13^C tracer experiments were carried out in the enclosures used for NCP measurements. A ^13^C labeled Na_2_CO_3_ solution (99.0% ^13^C) (Eurisotop, France) was added to each incubation plastic bag with a syringe. The solution was acidified underwater just before the injection to produce dissolved CO_2_ and HCO_3_^−^.

After incubation, *P. oceanica* shoots were uprooted; control plants were also collected. Isotopic and elemental measurements were performed with an isotopic ratio mass spectrometer (Isoprime 100, Isoprime, United-Kingdom) coupled to a C–N–S elemental analyzer (Vario Micro, Elementar, Germany). The abundance of ^13^C in *P. oceanica* leaves and in epiphytes was expressed in atom 13C%, i.e., the proportion of ^13^C atoms relative to the total C atoms (^12^C + ^13^C). Two units are used to express the elemental composition: the C content which is expressed in mg C shoot^−1^ (in leaves and in epiphytes) and the C relative concentration which is expressed in percent relative to the total dry weight (% dw). We have adopted a very conservative approach to calculate the ^13^C in excess in the labeled *P. oceanica* and epiphyte. Classically, the natural ^13^C abundances in samples were subtracted from the measured ^13^C abundance in control plants. But, natural ^13^C abundance for epiphytes and leaves were set to average values measured on control shoots plus three times the standard deviation around this average in order to minimize the risk to confound labeling effect and natural isotopic variability. Therefore, calculated enrichments as low as +0.001 ^13^C atom% were regarded as real enrichments against natural ^13^C composition. Using the dry weight, the relative content of carbon (% DW) and the ^13^C atom% in excess, we have calculated for each sample the quantity of excess ^13^C in the sample (mg^13^C in excess per shoot) and have calculated the contribution of epiphyte and leaves biomass to this ^13^C in excess.

For biomass accumulation: Artificial Seagrass Units (ASUs) (PVC band of 1 cm width and 50 cm length with a float at the extremity and fixed on a post (Macreadie et al., 2010) were deployed into the meadow to estimate the epiphyte production. After 10 days, ASUs were collected. Each ASU was scrapped with a razor blade (Dauby and Poulicek, 1995), epiphytes were oven-dried at 60°C for 48 h and then weighed. This epiphyte biomass was converted into mg C shoot^−1^ (by measurements in the C–N–S elemental analyzer; Vario Micro, Elementar, Germany) and used to calculate the daily production per square meter of substrate.

### What are the right times of sampling to have the snapshots of transcriptome and proteome responses to C-stress and to light stress (both supersaturating and limiting irradiances)?

For molecular analyses it is essential to setup the post-harvest in a way that does not perturb the ambient conditions and that shortens the time interval between plant sampling and tissue fixing. Here, we set the time intervals as short possible: at least two divers and many operators were required for each sampling. Collected plants were placed in black sealed containers *in situ*, then attached to a lift bag and handed to the operators waiting for samples from the dock for shallow sampling or on the boat for deep sampling. Thanks to this organization, we scored the minimum times *from the sea to the lab* of 10 min from shallow sites, 15 min from 20 m depth and 20 min from 30 m depth. The selection of sampling times along the day were made according to the common assessment for *in field* analyses and based on light changes as detailed in Table 1.

#### Incorporation of genomics tools

To incorporate gene expression analysis in the study of the main metabolic pathways involved in carbon budget, two different approaches were taken.

First, total transcriptome profiling was obtained using an Illumina next generation sequencing platform (Illumina GAIIx) available at the Genomics Research Centre (CRA_GPG, Fiorenzuola D’Arda, PC, Italy). High quality total RNA was extracted from pulled leaves from four individual shoots, two of which were collected at 5 m depth (12.00 and 18.30) and the other two at 20 m depth (12.00 and 18.30). Four complementary DNA (cDNA) libraries were prepared and run in a single Illumina GAIIx plate. Fragments were assembled in longer consensus sequences (contigs) and the following bioinformatic analysis allowed the identification of expressed genes through the annotation of contigs against public databases. All ESTs obtained will be stored in a new database, which will be made available to the scientific community. ESTs from the four samples were compared in order to find differentially expressed genes at the two depths and in two different time points. The set of differentially expressed genes was related to the physiological performances of the plant in the different conditions, and will serve as the basis for selecting sets of environmental responsive genes.

Second, expression levels of a set of genes encoding for molecular components of the photosynthetic and respiration apparatus were evaluated by RT-qPCR. Candidate genes have been selected according to their role in the different phases and in the different compartments of both processes (Table 2). For photosynthesis, we selected as genes of interest two structural components of Photosystem I (PSI), three genes encoding for subunits of PSII, two genes encoding for antenna proteins for each of the two Light-Harvesting complexes (LHCI and LHCII), one component of the chloroplast electron transport chain (Ferredoxin), and the gene encoding for RuBisCO small subunit. In order to investigate the photo-protective capacities of *P. oceanica*, we analyzed also the expression levels of one of the two key enzyme of Xanthophyll Cycle. Among those, nine genes (Table 2) had already been utilized in Ruocco et al. (2012) for assessing gene expression along the bathymetric gradient in a *P. oceanica* population located in the Island of Ischia (Gulf of Naples, Italy).

**Table 2:** **List of genes analyzed for assessing gene expression through RT-qPCR**.

Metabolic pathway	Gene	Protein	Function
**PHOTOSYNTHESIS**			
PSII	psbA	D1	Photosystem II 32kDa thylakoid membrane protein.
			One of the two reaction center proteins of photosystem II
PSII	psbD	D2	Photosystem II 34kDa protein. One of the two reaction center proteins of photosystem II, it is needed for assembly of a stable PSII complex
PSII	PsbS	CP22	Photosystem II 22kDa protein, chloroplastic.
			Seems to be involved in non-photochemical quenching (NPQ)
PSI	PsaJ	PSI-J	Photosystem I reaction center subunit IX.
			May help in the organization of the psaE and psaF subunits
PSI	PsaG	PSI-G	Photosystem I reaction center subunit V, chloroplastic.
			Function not known
LHCI	CAB-6A	LHCI type I CAB-6A	Chlorophyll a-b binding protein, chloroplastic. The light-harvesting complex (LHC) captures and delivers excitation energy to associated photosystems
LHCI	LHCA4	LHCI type III CAB-4	Chlorophyll a-b binding protein, chloroplastic.
LHCII	CAB-151	LHCII type II CAB-151	Chlorophyll a-b binding protein, chloroplastic.
LHCII	LHCB4.2	LHCB4.2	Chlorophyll a-b binding protein, chloroplastic.
Chloroplastic electron transport chain	SEND33	Ferredoxin-1	Ferredoxins are iron-sulfur proteins that transfer electrons in a wide variety of metabolic reactions
Carbon dioxide fixation	SSU5B	RuBisCO small chain 5B	RuBisCO catalyzes the carboxylation of D-ribulose-1,5-bisphosphate and the oxidative fragmentation of the pentose substrate
Xantophyll cycle -photoprotection	ZEP	Zeaxanthinepoxidase	Zeaxanthin epoxidase plays an important role in the xanthophyll cycle and abscisic acid (ABA) biosynthesis
**RESPIRATION**			
Ubiquinol-cytochrome *c* reductase complex	FES1	Ubiquinol-cytochrome *c* reductase iron-sulfur subunit	Component of the ubiquinol-cytochrome *c* reductase complex (complex III or cytochrome *b*-*c*1 complex), which is a respiratory chain that generates an electrochemical potential coupled to ATP synthesis
Mitochondrial electron transport chain	COX5B	Cytochrome *c* oxidase subunit 5B	One of the nuclear-coded polypeptide chains of cytochrome *c* oxidase, the terminal oxidase in mitochondrial electron transport
Mitochondrial electron transport chain	AOX1A	Alternative oxidase1A	Catalyzes the cyanide-resistant oxidation of ubiquinol and the reduction of molecular oxygen to water. Increases respiration when the cytochrome respiratory pathway is restricted
Mitochondrial electron transport chain	SDH2-2	Succinate dehydrogenase [ubiquinone] iron-sulfur subunit 2	Iron-sulfur protein (IP) subunit of succinate dehydrogenase (SDH), involved in complex II of the mitochondrial electron transport chain and responsible for transferring electrons from succinate to ubiquinone (coenzyme Q)
Tricarboxylic acid cycle	CMDH	Malate dehydrogenase	Catalytic activity: (S)-malate + NAD + = oxaloacetate + NADH

*Both photosynthetic and respiratory genes are shown*. *Gene names based on best scoring hits obtained blasting sequences against the Swiss-Prot database*.

For respiration we considered, three genes coding for proteins involved in the mitochondrial electron transport chain, one gene coding for a protein part of the ubiquinol-cytochrome *c* reductase complex and one gene involved in the tricarboxylic acid cycle.

Gene expression of selected genes has been evaluated by RT-qPCR, in relation to the expression of one reference gene (L23) selected among those previously identified in *P. oceanica* (Serra et al., 2012). The analysis has been performed on three individual samples collected along daily cycles at three different depths (5, 20, and 30 m; Table 1). Leaf tissue was cleaned from epiphytes and immediately stored in RNAlater^®^ Tissue Collection (AMBION, life technologies) in order to prevent RNA degradation. Total RNA extraction was performed using 60–100 mg wet weight tissue, according to the Aurum™ Total RNA Mini Kit (BIO-RAD) manufacturer’s instructions. RNA quantity and quality was assured by NanoDrop (ND-1000 UV-Vis spectrophotometer; NanoDrop Technologies) and 1% agarose gel electrophoresis. 500 ng of each RNA sample, were retro-transcribed in cDNA on GeneAmp PCR System 9700 (Perkin Elmer), with the iScript™ cDNA synthesis kit (BIO-RAD), following the manufacturer’s instructions.

RT-qPCR reactions were performed in MicroAmp Optical 384-Well reaction plate (Applied Biosystem) with Optical Adhesive Covers (Applied Biosystem) in a Viia7 Real Time PCR System (Applied Biosystem) using Sybr Green as fluorescent detection chemistry. RT-qPCR amplifications were conducted in 10 μl reaction volumes containing 5 μl of Fast Start SYBR Green Master Mix (Roche), 1 μl of cDNA template and 0.7 pmol μl^−1^ of each primer. Thermal profile was obtained as follows: 95°C for 10 min, 40 times 95°C for 15 s and 60°C for 1 min, 72°C for 5 min. For determining the specificity of the reaction, the melting curve of each amplicon from 60 to 95°C was also detected. The expression levels of each target gene were determined with REST tool (Relative expression software tool) (Pfaffl et al., 2002). Statistical analysis was performed using GraphPad Prism version 4.00 for Windows (GraphPad Software, San Diego, CA, USA).

The same shoots have been utilized to perform proteomics and a number of different analyses involving the other approaches utilized in the present project (Figure 1).

#### Incorporation of proteomic tools

The key step for proteomic analysis of marine plants, that must integrate with genomics and physiology, is the careful screening of target organs or tissues in which will address the proteomic study; leaf tissue is the eligible biological sample in seagrasses because leaves drive primary metabolism, provide the water and ions uptake in the place of roots (Kraemer et al., 1997), their sampling is not destructive for plants (Gobert et al., 2012); protein extraction from adult leaf tissues gives best results in term of pattern reproducibility among the biological replicates than those from intermediate and young leaves belonging the same plants (Dattolo et al., submitted; Spadafora et al., 2008).

##### Protein extraction and electrophoresis

The next step is process the samples up to a step that allows the safe transport for subsequent molecular analyses. This is the final challenge. The multi-steps protocol we adopted to extract proteins allowed us to obtain anhydrous tissue powders in which the proteins are denatured and the proteolytic degradations are inhibited. Tissue samples (see Table 1) have been shipped in this shape to the home laboratory. Here, proteins were extracted from tissue powder and purified following the protocol optimized for *P. oceanica* leaves (Spadafora et al., 2008). Briefly, 1 g of mature leaf tissue, frozen in N2, was ground to a fine powder and dissolved in 20% aqueous TCA (3-chloro-acetic acid) with 1% proteases inhibitor PMSF (phenylmethylsulfonylfluoride), to eliminate contaminants and precipitate proteins from leaf tissue. The extracted proteins were then treated with a phenol solution to isolate and purify the proteins from non-protein substances. Protein samples from all samples were processed on 1D SDS-PAGE; the Laemmli buffer system was used to cast a 6% stacking gel and 12.5% resolving gel. After denaturation at 100°C for 3 min, proteins were resolved at constant 200 V in a Bio-Rad mini Protean II apparatus. Peptide bands were quantified using Quantity One software (Bio-Rad). For each lane, area, and density of bands were calculated. Band volume was the product of band area and density. After background subtraction, band volume was normalized as the percentage of the total volume of protein bands on the same lane. The normalized volume (NV) of single band on the multiple gels from single depth and among samples was calculated, which was reproducible with 90% accuracy.

##### Orbitrap-LC-MS/MS and protein identification

Gel slides from each SDS-PAGE were cut in six slices and digested enzymatically with trypsin. Tryptic peptides were analyzed by liquid chromatography-tandem mass spectrometry (LC-MS/MS) using a high resolution LTQ-Orbitrap spectrometer (Thermo). Chromatography separations were conducted on a Waters XBridge C18 column (300 μm I.D. × 100 mm length and 3.5 μm particle size), using a linear gradient from 5 to 90% ACN, containing 0.1% formic acid with a flow of 4 μl min^−1^, including the regeneration step, one run lasted 70 min. Acquisitions were performed in the data-dependent MS/MS scanning mode (full MS scan range of 250–1800 m z^−1^ followed by full MS/MS scan for the most intense ion from the MS scan).

This yielded *de novo* protein sequences suitable for database searching. At first, peptide sequences generated by mass spectrometry were searched using GPM software (Global Proteome Machine) against plant databases. Peptide sequences, that were not identify with the method above, were further searched to GPM website using X!Tandem algorithm against the local database sequences building with all available *P. oceanica* and *Z. marina* sequences found in NCBI, Uniprot, and DrZompo databases (see Dattolo et al., submitted).

##### 1-DE Free-label approach and relative quantification by spectral count

**Figure 4:**
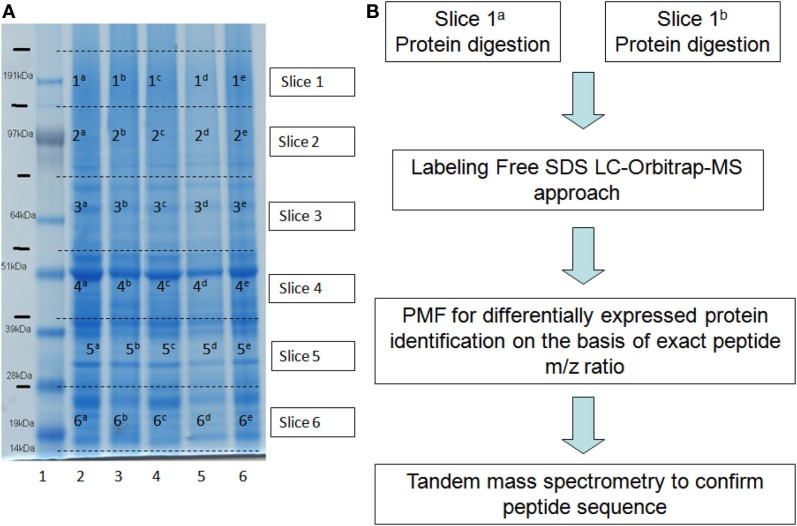
**(A)** 1-DE gel electrophoresis of leaf protein extracts from three depths. Dotted lines indicate each gel slice analyzed by labeling-free approach; Lane (1) markers; lane (2) 8.00 h, 3 m depth; lane (3) 8.00 h, 30 m depth; lane (4) 13.00 h, 3 m depth: lane (5) 13.30 h, 20 m depth; lane (6) 13.00 h, 30 m depth. **(B)** Experimental workflow applied to each pair of gel slices.

The Figure 4A shows the 1-DE-SDS separation of samples and the gel slices at ranges of molecular weight that were compared to detect differentially expressed proteins. The samples represent total protein extracts from *P. oceanica* adult leaves at different depths along the daily cycle. The differentially expression of proteins were evaluated by the labeling-free approach (Zhang and Wang, 2009). The workflow employed is described in the Figure 4B. After the proteins were digested with trypsin, the peptides obtained were analyzed Orbitrap-LC-MS/MS in singly charged ion production mode, and the peptide fingerprint was acquired using a high-mass-accuracy q-TOF instrument. In the spectral counting approach, relative protein quantification is achieved by comparing the number of identified MS/MS spectra from the same protein in each of the multiple LC-MS/MS datasets. This is possible because an increase in protein abundance typically results in an increase in the number of its proteolytic peptides, and *vice-versa*. This increased number of (tryptic) digests then usually results in an increase in protein sequence coverage, the number of identified unique peptides, and the number of identified total MS/MS spectra (spectral count) for each protein (Schulze and Usadel, 2010).

The differentially expressed peptides were analyzed using the fingerprint approach, and the differentially expressed proteins were then identified. The peptide sequences of the differentially expressed proteins were confirmed by MS/MS. Table 3 shows the differentially expressed proteins characterized in slices 4^a^ and 4^b^ corresponding to middle molecular weight peptides in the 1-DE-PAGE samples from −5 and −30 m at 13.00 h of same day. The identification score are reported with the peptide sequence and number of spectra are used to evaluate the level of protein expression. Many identified protein were found to be differentially expressed in this pair of gel slices; RuBisCo large subunits were about twofold over-expressed in shallow leaves relative to the deep leaves. However, all differentially expressed peptide among depths along daily cycle were detected by this approach.

**Table 3:** **Proteins detected in the slices 4^c^ and 4^d^ of the 1-DE (Figure 4) by means the label-free approach and spectral counting**.

log(e)	log(I)	% (Measured)	% (Corrected)	unique	spectra	Mr	Accession	Description
**(A)**								
-121	5.52	18	41	13	40	51.4	tr|B5WX89|B5WX89_9ARAE	Ribulose-biphosphate carboxylase (large subunit)
-82.2	5.45	18	49	7	30	54.6	tr|A0ARD7|A0ARD7_SMIRO	Photosystem II CP47 chlorophyll apoprotein
-45.7	5.4	16	39	5	25	51.8	tr|H2CPN3|H2CPN3_COLES	Photosystem II CP43 chlorophyll apoprotein
-40.9	5.15	22	34	4	15	37.6	EG_Contig15_1	Chlorophyll A-B binding protein (CAB)
-26.3	5.24	1.4	2	4	15	211.7	Zoma_B_i00191_2	Photosystem I P700 chlorophyll A apoprotein A1
-47.5	4.87	15	19	5	10	53.7	tr|Q4FGI4|Q4FGI4_TYPLA	ATP synthase subunit beta
-16.5	4.71	4.2	6	3	6	83.7	tr|Q8W0Q7|Q8W0Q7_SORBI	Methionine synthase protein
-28	4.58	8	15	3	6	55.1	sp|A6MMJ2|ATPA_DIOEL	ATP synthase subunit alpha, chloroplastic
-23.1	4.82	10	15	3	6	42.8	sp|P09315|G3PA_MAIZE	Glyceraldehyde-3-phosphate
								dehydrogenase A, chloroplastic
-19	4.82	4.5	7	2	5	88.2	Zoma_C_c61233_6	ATP synthase subunit alpha mitochondrial
-12	4	3.4	5	2	5	80	sp|A2YWQ1|HSP81_ORYSI	Heat shock protein 81-1
-19	4.34	7.2	9	3	5	59.1	sp|P19023|ATPBM_MAIZE	ATP synthase subunit beta, mitochondrial
-10.9	4.55	2.9	5	2	4	104.4	tr|Q43275|Q43275_ZOSMR	Putative plasma membrane H + -ATPase
-22.6	4	12	25	3	4	39.6	sp|Q4FFP4|PSBD_ACOAM	Photosystem II D2 protein
-8.9	4	6.2	23	2	4	38.9	sp|Q3V554|PSBA_ACOCL	Photosystem Q(B) protein
-12.7	4.47	15	25	2	4	24.2	sp|P05642|CYB6_MAIZE	Cytochrome *b*6
**(B)**								
-113	5.35	20	55	8	29	54.9	tr|H6TGJ9|H6TGJ9_9LILI	Photosystem II CP47 protein
-74	5.25	17	37	9	25	51.3	tr|B5WX64|B5WX64_9ARAE	Ribulose-biphosphate carboxylase
-46.9	5.3	6.3	14	5	19	158.1	Zoma_B_i08822_2	Photosystem II CP43 chlorophyll apoprotein
-49	4.95	35	53	5	17	37.6	EG_Contig15_1	Chlorophyll A-B binding protein (CAB)
-17	5	1.5	3	2	8	83.1	sp|A1EA08|PSAA_AGRST	Photosystem I P700 chlorophyll a apoprotein A1
-18.5	4	5.3	7	4	7	83.7	tr|Q8W0Q7|Q8W0Q7_SORBI	Methionine synthase protein
-7.9	4	2.6	3	1	5	93.9	tr|Q7XTK1|Q7XTK1_ORYSJ	Elongation factor
-8.1	4	6.2	23	2	5	38.9	sp|Q3V554|PSBA_ACOCL	Photosystem Q(B) protein
-18.5	4.47	1.7	3	3	4	240.7	Zoma_C_c64621_5	Photosystem I P700 chlorophyll a apoprotein A1
-26.9	4.63	4.3	9	2	4	54.7	tr|I1H3A2|I1H3A2_BRADI	Uncharacterized protein
-10.5	4	10	12	2	4	28.9	Pooc_Contig132_3	S-norcoclaurine synthase OS
-7.8	4.13	0.7	1	1	3	257	sp|B9FK36|ACC2_ORYSJ	Acetyl-CoA carboxylase 2
-26.8	4	5.9	6	1	2	39.6	sp|Q4FFP4|PSBD_ACOAM	Photosystem II D2 protein

*Statistical parameters and protein description are reported for shallow (A) and deep (B) plants. Proteins are sorted by number of spectra in both samples. The underline protiens are detected only in shallow or deep plants*.

*log(e): the base-10 log of the expectation that any particular protein assignment was made at random (*E*-value)*.

*log(I): the base-10 log of the sum of the fragment ion intensities in the tandem mass spectra used to make this assignment*.

*% (measured): the amino acid coverage of the protein in this assignment*.

*%(corrected): the amino acid coverage of the protein in this assignment/the coverage corrected for peptide sequences that are unlikely to be observed using normal proteomics methods*.

*Unique: the number of unique peptide sequences associated with this protein assignment*.

*Spectra: number of spectra assigned to the unique peptides for this protein*.

*Mr: the molecular mass of the entire protein sequence, in kiloDaltons*.

#### Incorporation of genetic tools

Meadows of *P. oceanica* have been extensively genotyped in the last few years overall Mediterranean Sea, using a set of 13 microsatellite markers. Variable levels of genetic diversity have been recorded, spanning from complete clonality (e.g., Ruggiero et al., 2002; Arnaud-Haond et al., 2012) to high diversity (e.g., Arnaud-Haond et al., 2007; Tomasello et al., 2009; Serra et al., 2010). Although the role of genetic and genotypic diversity of seagrass meadows on ecosystem functioning and on meadow resistance and resilience, have been debated in the recent literature (e.g., Ehlers et al., 2008; Arnaud-Haond et al., 2010), the assessment of genetic and genotypic variation, would allow to better evaluate factors underlying plasticity in the physiological response of the studied meadow. In order to do that, 20 samples from each of the two depths (5 and 20 m) were collected randomly and genotyped with the available putatively neutral microsatellite markers, as in Migliaccio et al. (2005). Allelic diversity, heterozygosity, and genotypic diversity have been compared between the two depths as well as between the STARESO meadows and other meadows at increasing distance from the study area. Levels of gene flow among meadows have also been assessed. Moreover, single shoots sampled for gene and protein expression analysis, and for photosynthesis and respiration measurements, have been genotyped, in order to investigate possible relationships between differences in physiological performances and difference in allelic composition of individual genotypes.

Shoots collected for the analysis with putatively neutral microsatellites markers, have also been genotyped with 51 EST-linked microsatellite (EST-msat) loci, following the protocol in D’Esposito et al. (in press). The EST-msat loci were assembled in four multiplex PCR reactions, capillary electrophoresis was performed in a Applied Biosystems 3730 DNA Analyzer and electropherograms were automatically scored using the software Peak Scanner (ABI). Search for outliers was performed comparing the two depths and other populations at variable distance from the study site. *Ad hoc* software was utilized and only loci positive to different statistical approaches were retained as real. Function of EST regions linked to outlier loci were evaluated in order to assess if depth has an effect on selecting genes related to carbon budget.

### Non-Invasive physiological analysis

Historically, many estimates of productivity of larger systems in nature has been done by extrapolating data from measurements from a small number of plants (or parts of plants) made in enclosures in the laboratory. However, these data have been shown to often yield values largely deviating from data obtained at more natural conditions. Also, the metabolic processes in plants are often linked to diel cycles, and thus often dramatically different at different times of the day, even if all environmental parameter might be similar. Thus it is important to follow these metabolic processes *in situ*, and during a longer time, as to be able to better estimate their true rates.

#### Mitochondrial respiration and photosynthesis

Surprisingly enough, there is a shortage of data on how much of the CO_2_ that is fixed through photosynthesis in seagrasses that are lost to the plant, or the system, by respiration. Seagrasses, like terrestrial plants, have both above- and below-ground tissues, making it much harder to measure rates of respiration of the underground roots and rhizomes, especially *in situ*. Therefore we are now incorporating this factor when measuring whole-plant or plant- community-based metabolism.

#### Incorporation of environmental sensors

Major obstacles in estimating community metabolism from physiological measurements on single plants are the scale in time and space. However, by the accurate measurement of key parameters, e.g., light and temperature, over the area and at different times of the day, and linking those to well-studied proxies for productivity, e.g., ETR, the scaling up of metabolic rates to meadow scale can be possible. Recent advances in automated fluorometery systems for *in situ* use (e.g., Shutter Fluorometer, Aquation, Australia) have enabled us to obtain regular measurements of both the effective quantum yield of photochemical energy conversion, PAR, and non-photochemical quenching. Using yield and PAR values we can calculate ETR and obtain a diel trace of ETR while avoiding artifacts due to transporting material away from the site of interest. The partitioning of non-photochemical quenching into several processes enabled by temporary dark-acclimation using the shutter provides additional insights into the nature of the physiological response to light over the course of a day.

Minilog TR temperature recorders, with a resolution of 0.2°C and an accuracy of ±0.3°C, were deployed at 3, 10, 20, and 30 m depth in canopy of the *P. oceanica* meadow (temporal data acquisition of every 30 min, GMT + 1).

**Figure 5:**
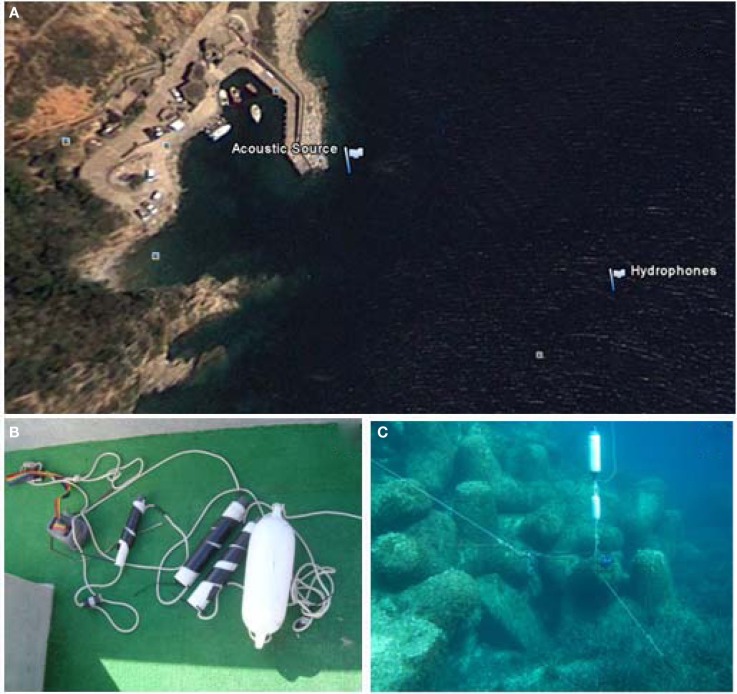
**Experimental area showing the location of the source and the hydrophones, (A) the source mooring and the Marsensing SR-1 self-recording hydrophones used in the underwater experiments (B,C)**.

**Figure 6:**
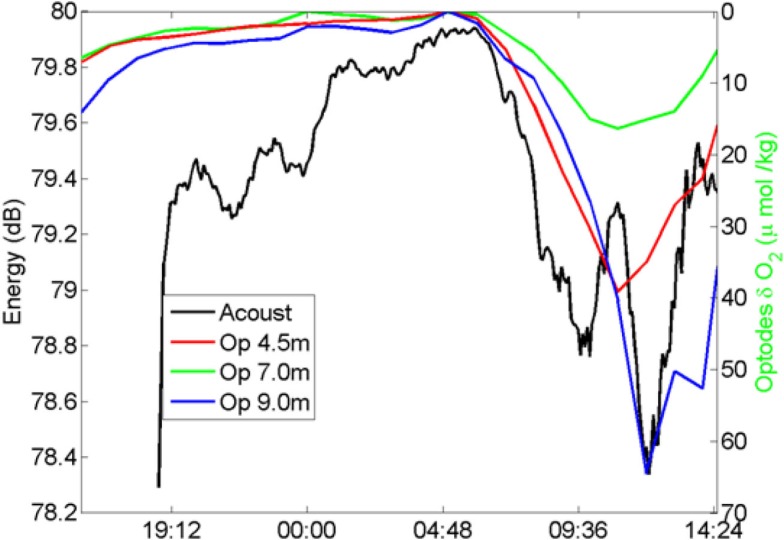
**Comparison between variability of the acoustic signal (energy) and the variability of dissolved O_2_ measured by optodes at three different depths**.

The acoustic signals were transmitted from a Lubell LL916C underwater speaker installed 2 m above the sea bottom in a site with water depth 8.5 m to three hydrophones Marsensing SR-1 moored in 21.5 m water column, 8, 4, and 2 m above the water column (Figure 5). The distance between the source and hydrophones mooring was ∼122 m. The acoustic data were acquired in two periods of about 2.5 days, separated by a bad weather event. The repetition rate of the signals was set to 15 min during the first period and 5 min in the second period to attain a higher time resolution. The signals were transmitted in three different frequency bands: low frequency band (400–800 Hz), medium frequency band (1500–3500 Hz), and frequency band (6500–8500 Hz). The instantaneous energy of the received signals and its half-hour running average was computed (Figure 6). We used the optode data from a mooring that has been deployed since August 2006 (Champenois and Borges, 2012). The mooring is located over the *P. oceanica* meadow at 10 m depth (8.733′E 42.567′N) close to the STARESO research station. Aanderaa O_2_ optodes (3835) mounted on Alec Instruments loggers were deployed at 4.5, 7.0, and 9.5 m depth. Oxygen saturation level and temperature were measured and logged at hourly intervals, to accommodate the typical duration of deployments (∼3 months) and lithium battery life at that sampling rate (∼5 months). Mooring data are interpreted in conjunction with other data, among which wind speed measured with an anemometer (Thies Clima) deployed on top of one of buildings of the STARESO station (at 11.8 m height) at a distance of about 100 m from the mooring. The temporal variations of the instantaneous energy of the accoustic signals and of the optodes was similar (Figure 6).

The time evolution of the received energy show high correlation with the photosynthesis activity: during the night the received energy is higher and its variability is low, during the daylight period the signal is highly attenuated with a remarkable fast fall of energy at sunrise. During daylight the variability of the received energy higher than during the night, it is observed an energy minimum at noon. Those results are in line with previous ones presented in works (Hermand and Alberotanza, 2000; Hermand, 2003; Wilson et al., 2012) and show the potential usage of acoustic method to track the integrated space-time variability of photosynthesis rate at community level.

## Expected Results

### How does mitochondrial respiration change over the day; is it changing in correlation with the light and in that case how?

The respiration changed with the time of day, following distinct diel cycles persistently over the 6 days the measurements was performed. The patterns of respiration were similar for the plants from the two depths, although slightly shifted in time. The respiration was always higher during the day, and the plants at the 20 m station had a peak in respiration around noon, while the plants from the 5 m station had their highest respiratory rates at around 15–18 h. Similarly, the lowest respiration for the 20 m plants was measured at six in the morning, while for the 20 m plants the lowest rates were at 9 h.

### What is the relative importance of plant respiration for the carbon budget of the meadow?

In general, irradiance (above canopy) peaked around noon, at about 900 μmol quanta m^−2^ s^−1^ at 5 m and at about 170 μmol quanta m^−2^ s^−1^ at 20 m. *In situ* ETR measurements peaked at these light levels, at 26.3 and 10.6^e−^ m^−2^ s^−1^, respectively. NCP peaked at around 8.6 μmol O_2_ m^−2^ s^−1^ at 3 m and at about 0.4 μmol O_2_ m^−2^ s^−1^ at 20 m. Then the respiration of the above-ground tissue was substantially higher than that of the below-ground tissue. As an average the above-ground tissue had a respiration rate 4.6 times higher than the rate of the below-ground tissue.

### How much do the epiphytic communities on the *P. oceanica* leaves contribute to the overall C-flux?

Change in biomass over the time is regularly used to estimate primary production in seagrasses. ASUs are simple and inexpensive and have the advantage of requiring minimal equipment. However, the ASU technique underestimates net productivity of epiphytes since it does not account for biomass losses due to excretion, decomposition, and harvest by grazers. ^13^C tracer incorporation into benthic chambers is simple but ASU and ^13^C tracer incorporation require sophisticated instrumentation for analytical measurements (C–N–S elemental analyzer and isotopic ratio mass spectrometer).

Seagrasses with a long life-span, such as *P. oceanica* support a complex community of epiphytic organisms and a multi-stratified community of diatoms and other microorganisms, crustose corallines, or crustose brown algae, sessile animals such as bryozoans, erect photophilous brown algae, and filamentous red algae (Van der Ben, 1971). The epiphyte community (species, biomass, algae vs. animals) and production is related to abiotic factors like light, water motion, temperature, nutriments and is related to biotic factors such as grazing. The epiphyte biomass is mainly related to substrate leaf availability, it decreases with increasing depth and increases from winter to summer (Lepoint et al., 1999). Light plays a strong role, the depth range restricting some epiphytic algae, in contrast, crustose corallines tolerate light-level variability and may colonize the entire length of *P. oceanica* leaves across the complete depth range of the meadow (Lepoint et al., 2007).

As the spatial structure of the epiphytic community occurs at different scales in relation to bathymetry (100 m), to meadow patchiness (10 m), to patch structure (1 m), and to the shoot itself (10 cm), we expected a large spatial variation in epiphytic community contribution to the overall C-flux. We also expect a day to day variability (only measurable by incubation approach) linked to light availability and to meteorological events. In the *P. oceanica* meadow, hourly epiphyte production is higher or similar to leaf production but epiphyte biomass accounted from 5 to 50% of the total above-ground biomass (Gobert et al., 2006) so epiphyte carbon assimilation ranges between 30 and 50% of the total *P. oceanica* shoot production (Modigh et al., 1998).

### How does the expression of specific genes and proteins change in relation to photosynthetic activity?

We expect changes in gene expression to be related to the amount of light available at the different depths during the daily cycles. The relationship between photosynthetic activity and efficiency, calculated by modulated fluorometry, and gene expression, obtained by RT-qPCR, can allow us to test the adaptive response of *P. oceanica* to different light regimes. We expect genes to be down-regulated with low-light. If this is the case, and in the presence of high photosynthetic efficiency at both depths, as suggested by previous unpublished PAM fluorometry data (Dattolo et al., submitted), we will confirm the plant to be shade adapted.

Results from genotyping of the two different stands, will allow us to infer the genetic isolation of plants along the depth gradient. This has already been found by Migliaccio et al. (2005), where plants sampled above and below the summer thermocline were found to be genetically isolated. The use of EST-related markers could allow the identification of putative outliers, which would result from positive or balancing selection acting between the two depths. Finally, we aim to relate inter-individual differences in gene expression with genotypic inter-individual differences.

### Can we correlate changes in productivity with changes in the transcriptome and in the proteome?

The main question is how good will be the correlations between gene expression and related protein levels, as the correlation vary depending on the system and should be as little as 40% (Vogel and Marcotte, 2012). There are many processes between transcription and translation and protein stability is a big factor. The half-life of different proteins can vary from minutes to days – whereas the degradation rate of mRNA would fall within a much tighter range, few hours for mRNAs vs. 48 h for protein (Vogel and Marcotte, 2012). Other factors include the lower rate of mRNA transcription compared to protein translation in cells, where single mRNAs transcribed per hour *versus* dozens of proteins/mRNA/hr. The biochemical diversity of proteins means that the individual correlation levels with the associated mRNA are going to vary a lot. We decided to consider, as a possible way to overcome the gap, the transcription level data; it can suggest whether or not the protein is present or not and roughly what level to expect to see the protein; i.e., a highly abundant protein will usually have a highly expressed mRNA. Therefore, the transcription data is useful for identifying potential candidates for follow-up work at the protein level and *vice-versa*.

Results obtained from RNA-Seq will allow the identification of differentially expressed sets of genes, extending the comprehension on the transcriptional regulation of *P. oceanica* in different environmental conditions. Identified regulatory networks and metabolic pathways will be correlated to the response to light and other environmental cues, allowing the identification of putative key genes in the physiological homeostasis of *P. oceanica*. Both RT-qPCR and RNA-Seq results will be correlated with changes in protein expression, in order to better identify regulatory networks and metabolic pathways mediating the response to light and depth.

We expect changes in proteins expression among primary metabolisms according to depth and daily light variations. This prospect is corroborated by a previous proteomic study on *P. oceanica*, in which RuBisCo was found to be 30% under-expressed in low-light acclimated leaves than those grown in high-light (Mazzuca et al., 2009). These findings indicated that light acclimation can affect the biochemical pathways of photosynthetic carbon assimilation. It is well known that during leaf development in land plants, lower levels of RuBisCo are closely tied to alterations in photosynthetic capacities which can strongly reduce the rate of leaf growth (Jiang and Rodermel, 1995). As a result of this impaired metabolism, there is a decrease in overall protein synthesis (Quick et al., 1991). In *P. oceanica* meadows, corresponding evidence between RuBisCo down-regulation, and decreased leaf length and shoot density were reported (Acunto et al., 2006). Interestingly, reduced leaf elongation was also observed in aquarium plants exposed to shading (Mazzuca, personal communication). These findings provide evidence that reductions in leaf growth may be related to decrease in primary production due the down-regulation of RuBisCo, both in plants acclimated to chronic low-light and in plants exposed to a short periods of shading. This is consistent with observations where leaves acclimated to chronic low-light exhibited lower protein synthesis, as indicated by lower protein yield in comparison to plants exposed to high-light conditions (Filadoro, 2007). We expect also variations in proteins related to photosystems functioning and structure among leaves acclimated to different depths; ultrastructural studies of *P. oceanica* chloroplasts showed that the exposure to chronic low-light drives the rearrangement between the two photosystems in a way that the PSI/PSII ratio is related to RuBisCo down-regulation; this may optimize daily carbon gains under low-light conditions (Mazzuca et al., 2009). The further independent study, whose partial results are reported here, confirmed the down-regulation of RuBisCo in leaves of deep plants; as shown in the Table 3, RuBisCo large subunit has counted higher spectra number in shallow samples than deep ones.

## Concluding Remarks

*Posidonia oceanica* meadows are complex ecosystems, whose dynamics, functioning, and evolution result from the interaction of numerous players, and from their response to environmental clues. No single actor plays independently nor is immune from the synergistic or antagonistic effects of the others. Seemingly, no single parameter can give a complete picture of the ecosystem and can be considered alone to fully describe the functioning and predict the fate of a seagrass meadow. The aim of this paper was to describe an integrative approach to the study of carbon cycling in *P. oceanica* meadows, supporting the concept that only a multidisciplinary study can uncover the emerging properties of an ecosystem that would otherwise remain undiscovered. We provide an evaluation of methods that measure the primary productivity of seagrasses, from the molecular (genomics, proteomics) and plant level (photosynthesis and respiration using carbon and oxygen flux techniques), to the community (net community metabolism and respiration) and ecosystem level (use of acoustics to measure oxygen production at large spatial scales and air-water CO_2_ flux).

## Conflict of Interest Statement

The authors declare that the research was conducted in the absence of any commercial or financial relationships that could be construed as a potential conflict of interest.
